# Impact of multi-vessel therapy to the risk of periprocedural myocardial injury after elective coronary intervention: exploratory study

**DOI:** 10.1186/s12872-017-0501-x

**Published:** 2017-02-27

**Authors:** Zhang-Wei Chen, Hong-Bo Yang, Ying-Hua Chen, Jian-Ying Ma, Ju-Ying Qian, Jun-Bo Ge

**Affiliations:** 1 Department of Cardiology, Shanghai Institute of Cardiovascular Diseases, Zhongshan Hospital, Fudan University, Shanghai, 200032 People’s Republic of China; 2 Department of Endocrinology, East Hospital, Tongji University, Shanghai, 200120 People’s Republic of China

**Keywords:** Coronary artery disease, Periprocedural myocardial injury, Percutaneous coronary intervention

## Abstract

**Backgrounds:**

Periprocedural myocardial injury (PMI) after elective percutaneous coronary intervention (PCI) significantly influences the prognosis of coronary artery disease (CAD). However, it was unclear whether the occurrence of PMI was associated with a series of controllable factors, such as PCI strategy or severity of CAD.

**Methods:**

A total of 544 consecutive stable CAD patients underwent elective PCI were enrolled. The main outcome is PMI, defined as troponin T after PCI was at least one value above the 99th percentile upper reference limit. Major adverse cardiac events (MACE), including all-cause death, repeat myocardial infarction and target vessel revascularization were record in the period of follow-up. Univariate and multivariate analysis was applied to assess predictors for the occurrence of PMI.

**Results:**

The incidence of PMI was 38.8% in the study. Compared with non-PMI patients (*n* = 333), PMI patients (*n* = 211) had more diseased vessels, higher Gensini and Syntax score. Meanwhile, there were higher incidence of MACE in PMI groups (9.5% vs. 3.2%, *P* < 0.01). We found that PMI patients underwent higher proportion of multi-vessel PCI simultaneously (32.2% vs. 10.5%, *P* < 0.01) and had more stents implanted (1.8 ± 0.8 vs. 1.4 ± 0.6, *P* < 0.01). Importantly, after simultaneously adjusted by other factors (such as age, diabetes, total cholesterol, number of diseased vessels, Gensini score and stent length), the risk of PMI was still increased 84% by multi-vessel PCI independently (OR = 1.654, 95% CI = 1.004–2.720, *P* < 0.05).

**Conclusions:**

The phenomenon of PMI occurred more commonly in stable CAD patients underwent multi-vessel PCI. Multi-vessel international therapy could increase the risk of PMI in elective PCI.

## Background

Cardiac troponin T, which is highly sensitive and specific biomarkers for myocardial injury [1], has been demonstrated to be associated with the prognosis of coronary artery disease (CAD). Increasing studies indicate that troponin elevation after percutaneous coronary intervention (PCI), which is relatively common after elective coronary intervention [2–5], has been considered as one of predictors for cardiac prognosis in CAD patients. It has also been demonstrated that even minor elevation in troponin T after elective PCI provides long-term prognostic information regarding mortality and myocardial infarction [5–8]. Therefore, it is critical for clinical practitioners to assess or find out a series of controllable factors or predictors for the occurrence of PMI, which could be useful to reduce the risk of PMI. Tandjung K found that number of implanted stents not stent type was associated with the incidence of PMI [9]. Prasad also confirmed that elevation of troponin T was not only associated with angiographic characteristics, but also provided long-term prognostic information [6]. However, there were few trials focused on multivariate analysis of the occurrence of PMI, and still fewer studies reported on the eastern population with PMI, especially in Chinese patients. Our previous study found that periprocedural myocardial injury (PMI) was associated with age, serum cholesterol and number of implanted stents [10]. Number of implanted stents was mostly determined by the severity of CAD during PCI procedure; therefore, we speculated that PMI might be partly impacted by severity of CAD or PCI strategy per se. In order to clarify this hypothesis in Chinese patients, we designed this study to demonstrate the clinical and PCI-related risk factors of PMI, meanwhile, to clarify whether the occurrence of PMI was associated PCI strategy (such as stepwise PCI or multi-vessel PCI).

## Methods

### Study population

From October 2011 to June 2012, a total of 544 consecutive patients referred to our hospital with stable angina pectoris for elective PCI were enrolled. The inclusion criteria were: (1) patients with 18 to 85 years of age; (2) providing a complete clinical history; (3) underwent coronary stent implantation; (4) normal pre-procedural troponin T (below the 99th percentile upper reference limit (URL), <0.03 ng/ml [6]) and creatine kinase (CK)-MB (<23U/L). The exclusion criteria were as follows: (1) acute coronary syndrome; (2) elevated cardiac troponin T (≥0.03 ng/ml) and CK-MB (≥23U/L) before coronary intervention; (3) heart failure, cardiomyopathy, congenital heart diseases and heart valve diseases; (4) treated coronary lesion was chronic total occlusion; (5) recent surgery or trauma; (6) active chronic inflammation; (7) dysfunction of hematological and immunological system; (8) carcinoma or a condition treated with immunosuppressive agents. We provided a written informed consent form to participants in our study and explained the entire study procedure to each patient. This study and consent procedure were approved by our local ethics committee (Ethics Committee of Zhongshan Hospital affiliated to Fudan University), and were carried out in accordance with the principles of the Declaration of Helsinki. Consent for publication of these data was obtained from each patient when they were admitted in our hospital.

### Clinical and laboratory measurements

The clinical characteristics of all patients including age, gender, primary hypertension, diabetes, smoking history, hyperlipidemia were recorded. Blood pressure and heart rate admitted to hospital were also detected. Fasting blood samples before PCI were drawn to detect complete blood-cell counts and blood biochemistry.

High sensitive cardiac troponin T (hs-cTnT) was measured before and within 24 h (from 10 to 20 h) after PCI by immunoturbidimetry (Hitachi 7600-020 automatic biochemistry analyzer). The 99th percentile upper reference limit (URL) of hs-cTnT is 0.03 ng/ml (this cut-point had been used in several previous studies, such as Miller’s and Prasad’s studies [6, 11]). Patients with post-procedural troponin T ≥ 0.03 ng/ml were defined as PMI group (*n* = 211) and others were defined as non-PMI group (*n* = 333).

### Echocardiography

Echocardiography was performed in all patients using a Philips IE33 instrument (Philips, Netherlands) with a 2–3.5 MHz transducer (X3-1), while left ventricular ejection fraction (LVEF) were detected. Observers who detected LVEF were blinded to the results of coronary angiography and grouping.

### Coronary angiography and intervention strategy

Elective coronary angiography was performed in all patients after admission. A patient was considered to have CAD when a stenosed lesion resulting in a 50% or greater reduction in lumen diameter existed in at least one of the coronary arteries. The severity of CAD was evaluated by Gensini score and Syntax score [12, 13]. Gensini score, Syntax score, number of diseased vessels, diseased lesions, treated vessels and implanted stents were recorded by observers who were blinded to the results of laboratory testing and study grouping. Patients with Gensini scores of 20 or more were defined as having severe CAD, which was approximately equal to one stenosed lesion of 70% or more in the proximal left anterior descending artery. The characteristics of lesions were also recorded and classified according to ACC/AHA coronary lesion classification [14].

Prior to PCI, all patients received adequate loading doses of acetylsalicylic acid (300 mg) and clopidogrel (30 mg). The PCI procedure was performed via the femoral or radial access route. Interventional techniques and further treatment during PCI were chosen at the operators’ discretion and according to current standards. Multi-vessel PCI was defined as more than one target vessel PCI at this procedure.

### Clinical outcomes

The primary outcomes evaluated for the present analysis were major adverse cardiac events (MACE), which including all-cause death, fatal or nonfatal myocardial infarction (MI) and target vessel revascularization (TVR, any clinically driven repeat PCI or bypass surgery of the target vessel). The risk of target lesion revascularization (TLR) was also analyzed.

### Statistical Analysis

All statistical analyses were performed with SPSS software 19.0. Data were presented as the percentage or mean ± standard deviation (SD). *Chi*-square analysis was used to compare the frequency for categorical variables, and Student’s *t* or correction *t* tests were used to compare means for continuous variables. Correlation analysis (Spearman test) was performed to evaluate the correlations among serum level of post-procedural troponin T, Gensini score and stent length. Multivariable analysis (logistic) was performed to identify the independent risk factors for PMI, to clarify whether the occurrence of PMI was associated PCI strategy (such as stepwise PCI or multi-vessel PCI). All *P*-values were two-sided, and *P* < 0.05 was considered to indicate statistical significance.

## Results

### Population baseline characteristics and clinical outcomes

A total of 544 stable CAD patients underwent elective PCI were enrolled in this study from October 2011 to June 2012. There were 378 men (69.5%) and 166 women (30.5%). The prevalence of hypertension, diabetes, and hyperlipidemia were 67.2% (366 patients), 25.2% (137 patients) and 31.8% (173 patients), respectively. Elevation of post-procedural troponin T observed in 211 patients (38.8%) (Average troponin T level: 0.156 ± 0.291 ng/ml) were defined as PMI group. Patients with normal post-procedural troponin T were defined as non-PMI group (*n* = 333, average troponin T: 0.015 ± 0.021 ng/ml). Baseline clinical characteristics of these patients were shown in Table 1.

**Table 1:** Comparison of clinical and laboratory characteristics between patients with and without PMI

	Non-PMI (*n* = 333)	PMI (*n* = 211)	*P*
Clinical characteristics			
Male (%)	231 (69.4%)	147 (69.6%)	0.941
Age (year)	62.9 ± 9.1	66.4 ± 9.3	<0.01
Hypertension (%)	215 (64.6%)	151 (71.6%)	0.090
Hyperlipidemia (%)	102 (30.6%)	71 (33.6%)	0.674
Diabetes (%)	73 (22.0%)	64 (30.3%)	0.028
Smoking (%)	140 (41.4%)	73 (34.6%)	0.088
LVEF (%)	62.4 ± 6.7	60.3 ± 5.8	0.542
Systolic blood pressure (mmHg)	127.8 ± 13.8	133.2 ± 15.9	0.105
Diastolic blood pressure (mmHg)	76.3 ± 8.3	75.2 ± 8.1	0.544
Heart rate (beat per minute)	69 ± 11	70 ± 8	0.802
Laboratory characteristics			
eGFR (ml/min)	94.4 ± 20.4	92.6 ± 22.6	0.486
Total cholesterol (mmol/L)	3.98 ± 1.02	4.26 ± 1.44	0.018
Triglyceride (mmol/L)	1.85 ± 0.96	2.04 ± 1.89	0.102
Pre-procedural CK-MB (U/L)	12.4 ± 5.8	13.9 ± 4.7	0.584
Post-procedural CK-MB (U/L)	18.5 ± 10.4	14.2 ± 5.6	0.054
Pre-procedure troponin T (ng/ml)	0.014 ± 0.018	0.016 ± 0.017	0.872
Post-procedure troponin T (ng/ml)	0.015 ± 0.021	0.156 ± 0.291	<0.01
Medication before angiography			
Beta-blocker (%)	273 (82.0)	177 (83.9)	0.794
ACEI/ARB (%)	229 (68.8)	137 (64.9)	0.588
Aspirin (%)	301 (90.4)	184 (87.2)	0.536
Statin (%)	314 (94.3)	200 (94.8)	0.921

*Abbreviations*: *ACEI/ARBs* angiotensin-converting enzyme inhibitors/angiotensin receptor blocker, *CK* creatine kinase, *eGFR* estimated glomerular filtration rate, *LVEF* left ventricular ejection fraction, *PMI* Periprocedural myocardial injury

The median period of follow-up in our study was 18.5 months. During the period of follow-up, 441 patients (81.0%) completed the clinical follow-up. The outcome of MACE was significantly higher in PMI group than that in non-PMI group (9.5% vs. 3.2%, *P* < 0.01). We also found that the incidence of TLR was higher in PMI groups (5.4% vs. 1.7%, *P* < 0.01) (Table 2).

**Table 2:** Long-term Follow-up data of this study (median period: 18 months)

	Non-PMI (*n* = 271)	PMI (*n* = 170)	*P*
MACE (%)	9	16	<0.01
TVR (%)	7	12	<0.01
Repeat MI (%)	2	3	0.386
All cause death (%)	0	1	0.616
TLR	5	9	<0.01

*Abbreviations*: *MACE* major adverse cardiac events, *MI* fatal or nonfatal myocardial infarction, *TLR* target lesion revascularization, *TVR* target vessel revascularization

### Angiographic and procedural characteristics

There were 1032 diseased vessel in these 544 patients. A total of 653 target vessels (1.2 target vessel per patient) were implanted with drug-eluting stent (DES). Angiographic characteristics were described in Table 3. Compare with non-PMI patients, PMI patient suffered from more severe CAD with higher Gensini score (43.3 ± 26.1 vs. 30.6 ± 20.0; *P* < 0.01), higher Syntax score (12.7 ± 6.9 vs. 16.9 ± 9.0, *P* < 0.01), more diseased vessels (2.2 ± 0.9 vs. 1.7 ± 0.8; *P* < 0.01) and more diseased lesions (3.6 ± 2.0 vs. 2.5 ± 1.6; *P* < 0.01). They also had more type C lesions than those in non-PMI group (Table 3).

**Table 3:** Comparison of Angiography and PCI characteristics between patients with and without PMI

	Non-PMI (*n* = 333)	PMI (*n* = 211)	*P*
Angiography characteristics			
Number of diseased vessels	1.7 ± 0.8	2.2 ± 0.9	<0.01
Multi-vessel stenosis (%)	166 (49.8%)	157 (74.4%)	<0.01
Number of diseased lesions	2.5 ± 1.6	3.6 ± 2.0	<0.01
Average Gensini score	30.6 ± 20.0	43.3 ± 26.1	<0.01
Average Syntax score	12.7 ± 6.9	16.9 ± 9.0	<0.01
Severe CAD (%)	237 (71.2%)	194 (91.9%)	<0.01
Target lesion characteristics	--464 lesions	--357 lesions	
A (%)	86 (18.5%)	53 (14.8%)	<0.01
B1 (%)	106 (22.8%)	64 (17.9%)	<0.01
B2 (%)	110 (23.7%)	90 (25.2%)	0.048
C (%)	162 (34.9%)	150 (42.0%)	<0.01
PCI characteristics			
Number of target vessels	1.1 ± 0.4	1.3 ± 0.5	<0.01
Multi-vessel PCI (%)	35 (10.5%)	68 (32.2%)	<0.01
Number of implanted stents	1.4 ± 0.6	1.8 ± 0.8	<0.01
Total stent length (mm)	34.7 ± 17.8	49.4 ± 25.9	<0.01
Type of stents			
--Sirolimus stent (%)	166 (49.8)	98 (46.4)	0.224
--Everolimus stent (%)	134 (40.2)	84 (40.0)	0.842
--Others (%)	33 (10.0)	29 (13.6)	0.218
Max inflation pressure (atm)	13.3 ± 2.6	13.0 ± 1.7	0.762
Contrast volume (ml)	144.8 ± 47.3	172.3 ± 63.5	<0.01
Procedural complications			
No reflow/slow reflow	4 (1.2%)	2 (1.0%)	0.682
Side-branch occlusion (%)	10 (3.0%)	12 (5.7%)	0.064
Distal thrombosis	1 (0.3%)	1 (0.5%)	0.342

*Abbreviations*: *PCI* Percutaneous coronary intervention, *PMI* Periprocedural myocardial injury

As concern to PCI characteristics (shown in Table 2), we found that PMI patients had higher proportion of multi-vessel PCI (32.2% vs. 10.5%, *P* < 0.01), more implanted stents (1.8 ± 0.8 vs. 1.4 ± 0.6, *P* < 0.01) and longer total length of implanted stents (49.4 ± 25.9 vs. 34.7 ± 17.8, *P* < 0.01).

### PMI associated with severity of CAD

**Fig. 1:**
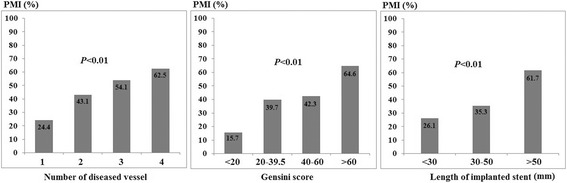
Incidence of PMI in patients stratified by severity of CAD

In order to clarify the association between occurrence of PMI and the severity of CAD, subgroups analysis was also performed in our study (Fig. 1). After stratified by the number of diseased vessels, extent of Gensini score and total length of implanted stents, we found that the incidence of PMI was significantly increased in more severe CAD patients.

### Effect of Multi-vessel PCI on PMI

**Fig. 2:**
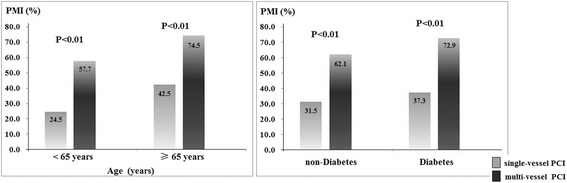
Incidence of PMI in multi-vessel and single-vessel PCI patients stratified by age and diabetes

**Fig. 3:**
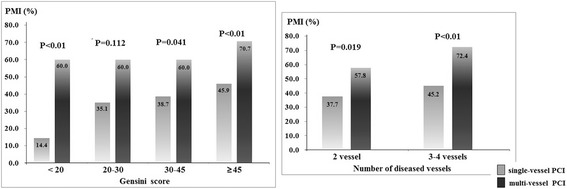
Incidence of PMI in multi-vessel and single-vessel PCI patients stratified by severity of disease

**Fig. 4:**
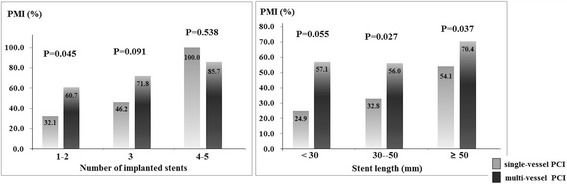
Incidence of PMI in multi-vessel and single-vessel PCI patients stratified by stent length and number

In this study, we found that patients with multi-vessel PCI simultaneously underwent higher proportion of PMI than those in single-vessel PCI group (66.0% vs. 32.8%, *P* < 0.01). In order to clarify the independent effect of multi-vessel PCI on PMI, we re-analyzed the effect of multi-vessel PCI after stratified patients by other factors, such as age, diabetes, Gensini score, number of diseased vessel, number of stents and total stent length. Multi-vessel PCI significantly increased the incidence of PMI in each sub-group analysis (Figs. 2, 3 and 4). Stratified by number of diseased vessel, patients with multi-vessel PCI had higher risk of PMI (2-vessel group: 57.8% vs. 37.7%; 3–4 vessel group: 72.4% vs. 45.2%, *P* < 0.01). While stratified by similar length of stents, patients with multi-vessel PCI also had higher prevalence of PMI (<30 mm stent length: 57.1% vs. 24.9%; 30–50 mm stent length: 56.0% vs. 32.8%; >50 mm stent length: 70.4% vs. 54.1%, *P* < 0.05).

Meanwhile, univariate analysis and multivariate logistic analysis were used to evaluate independent effect of multi-vessel PCI on the risk of PMI. In this analysis, PMI was adjusted by age, diabetes, cholesterol, number of diseased vessels, Gensini score, number of implanted stents and stents length. No matter adjusted by single factor or together, the risk of PMI was increased independently by multi-vessel PCI (OR = 1.654, 95% CI = 1.004–2.720, *P* < 0.05) (Tables 4 and 5).

**Table 4:** Odds ratios of patient-oriented and PCI-oriented factors for PMI (univariate analysis)

Adjusted by	OR	95% confidence intervals	*P*
Patient-oriented factors			
Age	1.043	1.023–1.063	<0.01
male	1.014	0.697–1.475	0.941
Diabetes	1.551	1.048–2.295	0.028
Hypertension	1.381	0.950–2.008	0.091
Total cholesterol	1.322	1.022–1.711	0.018
Triglyceride	1.150	0.940–1.408	0.102
PCI-oriented factors			
Gensini score	1.026	1.017–1.034	<0.01
Number of diseased vessels	1.867	1.516–2.298	<0.01
Number of implanted stents	2.329	1.805–3.005	<0.01
Total stent length	1.032	1.023–1.041	<0.01
Multi-vessel PCI	4.092	2.588–6.469	<0.01

*Abbreviations*: *PCI* Percutaneous coronary intervention, *PMI* Periprocedural myocardial injury

**Table 5:** Odds ratios of multivessel PCI for PMI adjusted by several factors (multivariate logistic analysis)

Adjusted by	OR	95% confidence intervals	*P*
Multi-vessel PCI (not adjusted)	4.092	2.588–6.469	<0.01
Age	4.224	2.649–6.734	<0.01
Diabetes	3.965	2.503–6.282	<0.01
Total cholesterol	3.136	1.829–5.629	<0.01
Gensini score	2.809	1.717–4.595	<0.01
Number of diseased vessels	2.750	1.667–4.537	<0.01
Number of implanted stents	2.167	1.249–3.758	<0.01
Total stent length	2.323	1.330–3.745	<0.01
Adjusted by all these factors	1.654	1.004–2.720	0.045

*Abbreviations*: *PCI* Percutaneous coronary intervention, *PMI* Periprocedural myocardial injury

## Discussion

As we know, PCI significantly improved the symptoms and prognosis in CAD patients. However, PMI, which occurred approximately 30% of patients after elective PCI, has been one of critical problems [15, 16]. Several previous studies documented that there was a significant association between cardiac adverse prognosis and PMI [6–8, 17, 18]. Therefore, another important thing is to find out why and when PMI happens or what its risk factors are.

Many prospective studies found that PMI patients always combined with more severe coronary disease, higher serum lipid level and more common clinical complications [6, 10]. Prasad also reported that post-procedural elevation of cardiac troponin was associated with multi-vessel PCI. However, there was few multivariate analysis applied between occurrence of PMI and its risk factors. Multi-vessel disease must be associated with severity of CAD, which could be demonstrated by severity score. Therefore, in order to eliminate the confounding impact of disease severity and stent length (which was related to PCI approach), multivariate logistic analysis was applied. As we showed in Table 4, not only patient-oriented factors, but also severity score, stents’ length and multi-vessel PCI approach were adjusted by logistic analysis together. We found that even adjusted by disease severity and stent length, multi-vessel PCI still increase the risk of PMI independently. In previous studies [6], there was also significant difference of multi-vessel PCI rate between PMI and non-PMI groups; however, it was only presented by univariate analysis. As we know, multi-vessel PCI was not only determined by multi-vessel disease and severe stenosis in each vessel, but also presented as different numbers of stents and different lengths of stents implantation. Therefore, multivariate analysis was performed in our study, while severity of CAD, numbers of stents and length of implanted stents were controlled simultaneously.

In this study, Gensini score, Syntax score, number of diseased vessel and number of implanted stents were applied to evaluate the severity of CAD. We found that occurrence of PMI was increased significantly in more severe CAD patients (Fig. 1). It has been documented that the most common mechanisms for PMI are distal embolization or side branch occlusion [16, 19]. Vessel lumen enlargement after stenting implantation probably results in a combination of plaque compression, plaque extrusion, plaque redistribution and micro-vessel embolization [20]. Severe stenosed lesions, with large lipid or necrotic core plaque, are at high risk of microembolization during the procedure of lesion enlargement and stents implantation [21]. Therefore, it is inevitable for PCI to result in different extent of microembolization. Post-procedural troponin T elevation may be a marker for severe atherosclerosis, increased plaque burden [22], presence of vulnerable plaques, endothelial dysfunction, microvascular injury, and inflammation [23].

Besides the severity of CAD, detailed PCI procedure was also compared between PMI and non-PMI groups. As we know, even among patients with the same number of PCI vessels, they still presented as different number and total length of implanted stents. Therefore, the association between multi-vessel PCI and higher prevalence of PMI might be confounded by number of stents and length of stents. In order to clarify the independent effect of multi-vessel PCI on PMI occurrence, we re-evaluated the effect of multi-vessel PCI in subgroup and multivariable analyses. When patients were stratified by number of implanted stents and stents length, incidence of PMI was increased significantly in multi-vessel PCI group. Most importantly, after analyzed by multivariate analysis, multi-vessel PCI was independently increased the risk of PMI.

We should note some of our study’s limitations. First, the number of included patients was small size. Second, it was a retrospective study, and its retrospective and non-randomized nature limited its potency to clarify the association between PMI and follow-up data. Third, present study did not include routine intravascular ultrasound, which was quite accuracy for lesion characteristic evaluation. These limitations will be taken into account in our further clinical researches and prospective studies.

## Conclusions

The phenomenon of PMI occurred more commonly in stable CAD patients underwent multi-vessel PCI. Multi-vessel international therapy could increase the risk of PMI in elective PCI.

## Abbreviations

ACEI/ARBs: Angiotensin-converting enzyme inhibitors/angiotensin receptor blocker
CAD: Coronary artery disease
CK: Creatine kinase
eGFR: Estimated glomerular filtration rate
LVEF: Left ventricular ejection fraction
MACE: Major adverse cardiac events
MI: Myocardial infarction
OR: Odds ratio
PCI: Percutaneous coronary intervention
PMI: Periprocedural myocardial injury
TLR: Target lesion revascularization
TVR: Target vessel revascularization
